# 

**Published:** 1901-12

**Authors:** 


### BOOKS RECEIVED.

A Text-Book of Medicine. For students and practitioners. By Dr. Adolf Strümpell, Professor and Director of the Medical Clinique at the University of Erlangen. Third American edition. Translated by permission from the thirteenth German edition, by Herman F. Vickery, A.B., M.D., Instructor in Clinical Medicine, Harvard University, etc., and Philip Coombs Knapp, A. M., M. D., Clinical Instructor in Diseases of the Nervous System, Harvard University, etc. With editorial notes by Frederick C. Shattuck, A. M., M. D., Jackson Professor of Clinical Medicine, Harvard University, etc. Octavo, pp. 1264. 185 illustrations and 1 plate. New York: D. Appleton & Co. 1901. [Price, cloth, $6.00; leather, $7.00.]

Transactions of the State Medical Association of Texas. Thirty-third Annual Session, held at Galveston, Texas, April 23, 24, 25 and 26, 1901. H. A. West, M. D., Secretary.

The Practice of Obstetrics. By American authors. Edited by Charles Jewett, M. D, Professor of Obstetrics and Gynecology in Long Island College Hospital, Brooklyn, N. Y. New (2d) edition, revised and enlarged. In one handsome 8vo volume of 775 pages, with 445 engravings in colors and black, and 35 full-page colored plates. Philadelphia and New York: Lea Brothers & Co. 1901. [Price, cloth, $5.00 net; leather, $6.00 net; half-morocco, $6.50 net.]

A Text-Book of Physiological Chemistry. For students of medicine and physicians. By Charles E. Simon, M. D., of Baltimore, Author of "Simon's Clinical Diagnosis," etc In one 8vo volume of 452 pages. Philadelphia and New York: Lea Brothers & Co. 1901. [Price, cloth, $3.25 net.]

Anatomy, Descriptive and Surgical. By Henry Gray, F. R. S., Lecturer on Anatomy at St. George's Hospital, London. Thoroughly revised American from the 15th English edition. In one imperial 8vo volume of 1246 pages, with 780 illustrations. Philadelphia and New York: Lea Brothers & Co. 1901. [Price, with illustrations in black, cloth, $5.50 net; leather, $6.50 net. Price, with illustrations in colors, cloth, $6.25 net; leather, $7.25 net.]

Text Book of Nervous Diseases. Being a compendium for the use of students and practitioners of medicine. By Charles L. Dana, A. M., M. D., Professor of Nervous Diseases in Cornell University Medical College; Visiting Physician to Bellevue Hospital; Neurologist to the Montefiore Hospital. Fifth edition. Octavo, pp. 646. 244 illustrations. New York: William Wood & Co. 1901. [Price, $3.50.]

A Text-Book on Diseases of the Ear, Nose and Throat. By Charles H. Burnett, M. D., E. Fletcher Ingals, M. D., James E. Newcomb, M. D. Octavo, pp. 734. Illustrated. Philadelphia and London; J. B. Lippincott Co. 1901. [Price, $5.00.]

A System of Physiologic Therapeutics. A practical exposition of the methods, other than drug-giving, useful in the prevention of disease and in the treatment of the sick. Edited by Solomon Solis Cohen, A. M., M. D., Professor of Medicine and Therapeutics in the Philadelphia Polyclinic; Lecturer on Clinical Medicine at Jefferson Medical College; Physician to the Philadelphia Hospital, etc. Volume III.—Climatology: Health Resorts, Mineral Springs. By F. Parkes Weber, M. A., M. D., F. R. C. P. (Lond.), Physician to the German Hospital, Dalston; Assistant Physician North London Hospital for Consumption, etc. With the collaboration for America of Guy Hinsdale, A. M., M. D., Secretary of the American Climatological Association, etc. In two books. Book I.—Principles of Climatotherapy, Ocean Voyages, Mediterranean, European and British Health Resorts. Book II.—Mineral Springs, Therapeutics, etc. Illustrated with maps. [Price for the complete set, $22.00 net.]

American Edition of Nothnagel's Encyclopedia. Typhoid and Typhus Fevers. By Dr. H. Curschmann, of Leipzig. Edited, with additions, by William Osler, M. D., Professor of the Principles and Practice of Medicine, Johns Hopkins University. Handsome 8vo of 646 pages. Illustrated; including a number of valuable temperature charts and two full-page colored plates. Philadelphia and London: W. B. Saunders & Co. 1901. [Cloth, $5.00 net; sheep or half-morocco, $6.00 net.]

An American Text-Book of Pathology. Edited by Ludvig Hektoen, M. D., Professor of Pathology, Rush Medical College, Chicago; and David Riesman, M. D., Professor of Clinical Medicine, Philadelphia Polyclinic. Handsome imperial 8vo of 1245 pages, 443 illustrations, 66 of them in colors. Philadelphia and London: W. B. Saunders & Co. 1901. [Cloth, $7.50; sheep or half-morocco, 8.50 net.]

A Reference Handbook of the Medical Sciences. Embracing the Entire Range of Scientific and Practical Medicine and Allied Science. By various writers. A new edition, completely revised and rewritten. Edited by Albert H. Buck, M. D., New York City. Volume III. CHL–E.Q.U. Illustrated by chromo-lithographs and 676 half-tone and wood engravings. Nine volumes, imperial 8vo. New York: William Wood & Co. 1901. [Price, muslin, $6.00 per volume; leather, $7.00 per volume; half-morocco, $8 00 per volume.]

A Treatise on Surgery by American Authors. For students and practitioners of medicine and surgery. Edited by Roswell Park, M. D., Professor of Surgery in the University of Buffalo, N. Y. New (3d) edition. In one royal 8vo volume of 1350 pages, with 692 engravings and 64 full-page plates in colors and monochrome. Philadelphia and New York: Lea Brothers & Co. [Cloth, $7.00 net; leather, $8.00 net.]

A Text-Book of Surgery. By Dr. Hermann Tellmans, Professor in the University of Leipsic. Translated from the seventh German edition by Benjamin T. Tilton, M. D., and John Rogers, M. D., Instructors in Surgery, Cornell University. Edited by Lewis A. Stimson, M. D., Professor of Surgery, Cornell University. Volume I. The Principles of Surgery and Surgical Pathology. With 516 illustrations. Pp. viii.—841. New York: D. Appleton and Co. 1901.

International Clinics. A Quarterly of Clinical Lectures and Especially Prepared Articles on Medicine, Neurology, Surgery, Therapeutics, Obstetrics, Pediatrics, Pathology, Dermatology, Diseases of the Eye, Ear, Nose and Throat, etc. Edited by Henry W. Cattell, A.M., M.D., of Philadelphia, with the collaboration of John B. Murphy, M. D., of Chicago; Alexander D. Blackader, M. D., of Montreal; H. C. Wood, M. D., of Philadelphia; T. M. Rotch, M. D., of Boston; E. Landolt, M.D , of Paris; Thomas G. Morton, M.D., and Charles H. Reed, M.D., of Philadelphia; J. W. Ballantyne, M.D., of Edinburgh, and John Harold, M D., of London. Volume III. Eleventh series. Philadelphia: J. B. Lippincott Co. 1901. [Price, cloth, $2.00 each; half-leather, $2.25 each.]

Infant-Feeding in its Relation to Health and Disease. By Louis Fischer, M. D., Visiting Physician to the Willard Parker and Reception Hospitals of New York City, etc. Duodecimo, pp. 351. 52 illustrations, with 23 charts and tables, mostly original. Second edition. Philadelphia: F. A. Davis Co. 1901.

The Standard Medical Manual. A Handbook of Practical Medicine. By Alfred S. Burdick, M. D., Junior Professor of Practice of Medicine, Illinois Medical College; Member of the American Medical Association, the Illinois State Medical Society, etc. Pages, 921. Illustrated. Chicago: G. P. Engelhard & Co. [Price, cloth, $4 00.]

A Treatise on the Acute, Infectious Exanthemata. Including Variola, Rubeola, Scarlatina, Rubella, Varicella and Vaccinia, with especial reference to Diagnosis and Treatment. By William Thomas Corlett, M. D., L. R. C. P., Lond., Professor of Dermatology and Syphilology in Western Reserve University; Physician for Diseases of the Skin to Lakeside Hospital; Consulting Dermatologist to Charity Hospital, St Alexis Hospital and the City Hospital, Cleveland; Member of the American Dermatological Association and the Dermatological Society of Great Britain and Ireland. Illustrated by 12 colored plates, 28 half-tone plates from life, and 2 engravings. Pp. viii.—392 Sold only by subscription. Philadelphia: F. A. Davis Co. 1901. [Price, cloth, $4.00 net, delivered.]

A Text-Book of Pharmacology. Including Therapeutics, Materia Medica, Pharmacy, Prescription-Writing, Toxicology, etc. By Torald Sollmann, M. D., Assistant Professor of Pharmacology and Materia Medica, Western Reserve University, Cleveland, Ohio. Royal 8vo volume of 880 pages, fully illustrated. Philadelphia and London: W. B. Saunders & Co. 1901. [Price, cloth, $3.75 net.]

A Laboratory Handbook of Physiologic Chemistry and Urine-examination. By Charles G. L. Wolf, M. D., Instructor in Physiologic Chemistry, Cornell University Medical College, New York. Duodecimo volume of 190 pages, fully illustrated Philadelphia and London: W. B. Saunders & Co. 1901. [Price, cloth, $1.25 net.]

Saunders's Medical Hand-Atlases. Atlas and Epitome of Bacteriology. A Text-Book of Special Bacteriologic Diagnosis. By Professor Dr. K. B. Lehmann, Director of the Hygienic Institute in Würzburg; and R. O. Neumann, Dr. Phil and Med., Assistant in the Hygienic Institute in Würzburg. From the second enlarged and revised German edition. Edited by George H. Weaver, M. D., Assistant Professor of Pathology, Rush Medical College, Chicago. In two volumes. Part I., consisting of 632 colored figures on 69 lithographic plates. Part II., consisting of 511 pages of text, illustrated. Philadelphia and London: W. B. Saunders & Co. 1901. [Price, cloth, $5.00 net.]

Libertinism and Marriage. By Dr. Louis Jullien (Paris), Surgeon of Saint-Lazare Prison; Laureate of the Institute, of the Academy of Medicine, and of the Faculty of Medicine of Paris. Translated by R. B. Douglas Pp. v.—169. Philadelphia: F. A. Davis Co. 1901. [Price, cloth, $1.00 net.]

Diseases of the Intestines. Their Special Pathology, Diagnosis and Treatment. With sections on anatomy and physiology, microscopic and chemic examination of the intestinal contents, secretions, feces and urine. Intestinal bacteria and parasites; surgery of the intestines; dietetics; diseases of the rectum, etc. By John C. Hemmeter, M. D., Philos D., Professor in the Medical Department of the University of Maryland; Consultant to the University and Director of the Clinical Laboratory, etc. In two volumes. Volume I Anatomy, Physiology, Intestinal Bacteria, Methods of Diagnosis, Therapy and Materia Medica of Intestinal Diseases, Diarrhea, Constipation, Enteralgia and Enterodynia, Meteorism, Dystrypsia, Enteritis, Colitis, Dysentery, Intestinal Ulcers, Intestinal Neoplasms, etc. With many original illustrations, some of which are in colors. Large 8vo, 740 pages. Philadelphia: P. Blakiston's Son & Co. 1901. [Price, $5.00 per volume.]

The Surgical Treatment of Disfigurements and Deformities of the Face. By John B. Roberts, A. M., M. D, Professor of Surgery in the Philadelphia Polyclinic; Surgeon to the Methodist Hospital. Duodecimo, pp. 72. Second edition, with a chapter on the Reconstruction of Syphilitic Noses. Sixty-two figures. Philadelphia: The Philadelphia Medical Publishing Co. 1901.

